# Effect of Steam Explosion (SE) Pretreatment on the Contamination of Woody Biomass with Metallic Inhibitors

**DOI:** 10.3390/ma18194536

**Published:** 2025-09-29

**Authors:** Jan Szadkowski, Anna Gałązka, Witold Jan Wardal

**Affiliations:** 1 Department of Wood Science and Wood Protection, Institute of Wood Sciences and Furniture, Warsaw University of Life Sciences—SGGW, 159 Nowoursynowska St., 02-787 Warsaw, Poland; 2 Faculty of Wood Technology, Warsaw University of Life Sciences—SGGW, 159 Nowoursynowska St., 02-787 Warsaw, Poland; anna.galazka1995@gmail.com; 3 Department of Technology and Entrepreneurship in Wood Industry, Institute of Wood Sciences and Furniture, Warsaw University of Life Sciences—SGGW, 159 Nowoursynowska St., 02-787 Warsaw, Poland

**Keywords:** X-ray fluorescence spectrometer, poplar, steam explosion, inhibitors, wood

## Abstract

The aim of this study was to check the content of metal inhibitors before and after the pre-treatment of fast-growing poplar wood using steam explosion (SE) at selected temperatures (160, 175, 190 and 205 °C). An X-ray fluorescence spectrometer (XRF) was used for the analysis. The material was analysed after pre-treatment and in its native form in two variants: incinerated wood chips and incinerated wood chips dissolved in nitric acid. The analysis was intended to show the difference in the content of metals inhibiting biological processes, including enzymatic hydrolysis and fermentation (i.e., chromium, manganese, iron, nickel, copper and zinc). The study aimed to identify changes in the content of metallic inhibitors depending on the SE temperature and to demonstrate differences depending on the methodology used to measure metals in the tested material. The greatest change in metal content in the material after pre-treatment was observed for pre-treatment at 175 °C, regardless of the determination method used. Both methods allow the trend in changes in metal content in wood material to be determined. However, due to the heterogeneous structure of wood, the methods give different results, especially for iron.

## 1. Introduction

The development of a zero-emission economy brings with it the need to develop technology that is efficient and as economically viable as possible, while ensuring that the environmental impact of the technology developed is minimal. One promising energy generation technology is the conversion of lignocellulosic biomass to liquid biofuels such as bioethanol [1]. Another method of obtaining energy feedstock in low-carbon biofuel technology is to gasify biomass and then use the resulting gas mixture for power generation or chemical synthesis [2,3].

Conversion can be carried out on any lignocellulosic biomass. Its yield will depend on its chemical composition, moisture content, and pretreatment process. The lignocellulosic material is one whose main cell wall building block is a lignocellulosic complex consisting of three basic substances: cellulose, hemicelluloses and lignin [4].

Depending on the raw materials used and the technology, biofuels are subdivided into three generations (there are three generations of biofuels where generation I fuels use lignocellulosic material such as wood or cereal grains for energy production (combustion) or to produce energy raw materials (ethanol), generation II are lignocellulosic materials that do not compete with the food industry or are production waste from other industries with no further use (straw, post-consumer wood). Generation III, which is referred to as the biomass of the future, is based on raw materials that are not used in other industries, are characterised by high growth and low environmental impact and include fuels derived from algae and trees genetically modified for rapid growth [5,6,7,8].

Efficient extraction of energy raw materials from lignocellulosic biomass requires the use of pretreatment, i.e., a process to increase the availability of chemicals for subsequent processes. Mechanical, chemical, physico-chemical and biological pretreatments are distinguished [9,10,11,12].

During the pretreatment and conversion of lignocellulosic materials, organic and inorganic chemical compounds are formed that can hinder the conversion of biomass in subsequent stages. They are inhibitors of biological processes, which are part of the basis of environmentally friendly technologies. Such compounds include, for example, furfural, hydroxymethylfurfural, organic acids (lactic acid, oleic acid, levulinic acid) mineral acids, e.g., carbonic acid, metal ions such as nickel, copper, iron, magnesium and chromium [7,12]. These compounds impair the action of enzymes, enzyme analogues and the organisms themselves used in biomass conversion, e.g., yeast used for ethanol production [9,12,13,14,15]. The toxicity of metals has a dual effect on living organisms, including the biological processes in which they are used. Metals do not degrade in the natural environment or in the reaction environment, which causes these compounds to accumulate in nature and in closed-loop biomass processing. Concentrations of 36 mg/L cadmium, 27 mg/L chromium, 8.9–20.7 mg/L copper, 35 mg/L nickel and 7.7 mg/L zinc inhibit the activity of methanogens by 50%. A noticeable decrease in methane production in the biogas plant is observed for concentrations above 0.16 mmol/L of copper, 0.17 mmol/L of nickel, 0.15 mmol/L of zinc and 0.05 mmol/L of lead [16]. With copper content ranging from 10.24 mg/L to 80.64 mg/L, a decrease in alcoholic fermentation efficiency and an increase in the time required for the fermentation process are observed [17].

Currently, there are no uniform methods for analyzing metals in lignocellulosic biomass. The analyses used in laboratories are based on the equipment available to scientists. Methods based on infrared spectroscopy, such as absorption spectroscopy (IR) or Fourier transform infrared spectroscopy (FTIR), scanning electron microscopy (SEM), energy dispersive X-ray spectroscopy (EDX), X-ray diffraction (XRD) analysis, electron spin resonance spectroscopy (ESR), nuclear magnetic resonance (NMR), X-ray photoelectron spectroscopy (XPS), X-ray absorption spectroscopy (XAS), and chemical fractionation analysis (CFA) [18,19,20].

These methods differ in their mechanism of action, accuracy, and sensitivity. Many methods are subject to errors related to the heterogeneous structure of biomass and contamination arising during the grinding of the test material [21,22,23].

There is a need to develop a method for analyzing the metal content in lignocellulosic biomass in order to standardize the determination methodology, which will contribute to more accurate measurements and enable the comparison of the obtained results. During the research, we attempted to develop a method for preparing material for analysis in order to obtain reliable, repeatable results independent of the imposed biomass structure.

Steam explosion (SE) is considered the most promising method of pretreatment in lignocellulosic biomass biofuel technology. The steam explosion method uses the action of saturated steam, water or ammonia and carbon dioxide at elevated temperature and pressure. The best-studied method to date is steam action. The steam explosion is carried out in a special high-pressure autoclave. The test material, sealed in the autoclave tank, is subjected to high temperature and high pressure. This is followed by rapid decompression to atmospheric pressure [24]. As a result of SE, lignin is degraded and cellulose and part of the hemicelluloses are broken down to simple sugar such as xylose, galactose, mannose, glucose. Material prepared in this way is more accessible to hydrolytic enzymes [25].

The steam explosion method is considered one of the most effective and efficient pretreatment methods. The process requires 70% less energy than physical methods [26]. Above that, it allows for a reduction in waste and recycling costs [27]. The difference in the cost of the steam explosion process between 160 °C and 205 °C estimated in 2021 was between USD 134.16 per tonne of biomass for the 160 °C process and USD 471. This is three times higher than the process at a lower temperature [15].

However, some of the hemicelluloses are degraded as a result of high temperatures. This is a limitation due to the loss of monosaccharides that would have been formed from their degradation. The effects of high temperature and pressure not only leach the mineral compounds contained in the wood but also oxidise the metal from the apparatus and cause corrosion. The released metal compounds penetrate the test material and, along with the decomposition products of the biomass components, constitute metal inhibitors that can hinder and slow down the enzymatic hydrolysis process. The number of inhibitors that entered the wood material depends on the conditions under which the steam explosion process was carried out. During the steam explosion experiment, care must be taken to select the appropriate process parameters. The temperature at which the steam explosion is carried out ranges from 160 °C to 290 °C. The pressure ranges from 5 to 35 bar, depending on the temperature and apparatus [28].

The aim of this study was to analyse the content of heavy metals in the wood of 5-year-old genetically modified poplar pretreated by steam explosion at selected temperatures. To realise the aim of the study, the contents of selected heavy metals were compared in ashed wood material pretreated by steam explosion at temperatures of 160–205 °C. Additional verification was provided by measurements made on ash samples dissolved in nitric acid.

## 2. Materials and Methods

### 2.1. Materials

Wood of fast-growing poplar (genetically modified MPK 4-11-2) five years after planting on the experimental site was used for the study (52°08′42″ N 21°04′07″ E). The harvested material was dried in a laboratory dryer to an absolute moisture content of 0%. After drying, the material was debarked, selected to remove defects such as knots, false heartwood, etc. The material thus selected was ground using a laboratory knife mill. The poplar chips thus obtained were separated into fractions using laboratory sieves. The 0.43–1.0 mm fraction was used for further analyses and experiments.

### 2.2. Steam Explosion (SE)

The steam explosion was performed on 20 g of 0.43–1.0 mm chips flooded with distilled water so that there was at least 10 mL of water per 1 g of dry chips. To remove air from the wood pores, the wood shavings were pre-poured in a beaker with 1/3 of the volume of water and heated at 90 °C with stirring for 20 min. The material thus prepared was placed in a stainless-steel high-pressure laboratory autoclave fitted with a 5 cm diameter spherical valve and a receiver in which a vacuum is set before sudden decompression of the autoclave. The autoclave and the valve are suitable for operation up to 250 °C. The autoclave itself has a built-in electric heating system. After placing the pre-soaked chips in the autoclave, water was added so that there was 12 g of distilled water per 1 g of wood material. The heating process was carried out without maintaining the process temperature (after reaching the set temperature, decompression followed). The experiment was conducted at four temperatures: 160, 175, 190 and 205 °C. After the steam explosion, the material was transferred to a receiver, from where it was carefully rinsed with distilled water into a glass beaker (2000 cm^3^). Using a tissue filter placed on a Büchner funnel (paper filter type 640 m, filtration time 27 s (retention > 4 µm). Medium-speed filtration) connected to a mechanical vacuum pump, the solid fraction was separated from the liquid fraction. Three experiments were carried out for each temperature variant [15].

### 2.3. Measurement of Heavy Metal Content

After steam explosion treatment, the material was ashed in a muffle furnace at 600 °C prior to metal content analysis. The muffle furnace dimensions are 440 mm × 495 mm × 530 mm. It is manufactured by SNOL industrial plant located in Plento G3, Narkūnai, 28104 Utenos r.sav., Lithuania. The heavy metal content was measured by fluorescence spectrometer from Spectro Mixed M (manufactured by SPECTRO Analytical Instruments GmbH, Boschstrasse 10, D-47533, Kleve, Germany). The technical specifications of this instrument, as provided by the manufacturer in the user manual, are as follows.

Excitation system:

- maximum power: 30 W
- maximum current: 0.8 mA
- maximum voltage: 50 kV
- X-ray tube with air-cooled molybdenum anode

The measurement chamber has a measurement table positioning accuracy of 2.5 μm. The chamber dimensions are 540 mm × 600 mm × 2500 mm. The image on the computer monitor is displayed using a dual camera system. The ashes obtained from the combustion were placed on a clean and appropriately labelled sheet of paper. The material was arranged so that the tops of the samples were on one level. The sheet with the ashes was transferred to the test table of the XRF spectrometer and an initial measurement was made.

Three measurement points were determined on each sample (using a 2 mm × 2 mm aperture). Each point was exposed for 300 s. The relative changes in the content of heavy metals such as chromium, iron, nickel, copper, manganese and zinc expressed as percentages were compared. In order to accurately verify the changes in the metal content of the steam-blasted material, an analysis of the metal content of ash dissolved in nitric acid was carried out. Such an assay allows measurement of the content of selected metals with greater accuracy due to the ability to accurately calibrate measurements of liquid samples. Samples of approximately 0.1 g of ash were weighed out on a laboratory balance. Volume 1 cm^3^ of concentrated nitric acid was added to each sample and boiled until dissolved. The ash dissolved after about 4 h of boiling. The solution thus obtained was then cooled and neutralised by adding 2 cm^3^ of ammonia water (25%). During neutralisation, indicator papers were used to ensure that the pH of the resulting solution did not exceed 7. The method was based on Zielenkiewicz’s research from 2015 [29], in which the calibration of the content of individual metals for XRF used in the testing of samples burned and dissolved in nitric acid was developed, which is reflected in earlier work [30].

During neutralisation, indicator papers were used to ensure that the pH of the obtained solution did not exceed 7.

Three drops (0.3 cm^3^) of the solution were tested from each sample. The drops were placed with a pipette on a dish to be placed on the measuring table of the spectrometer. One measuring point was determined on each drop. Each sample was exposed for 300 s. The process was repeated three times.

### 2.4. Statistical Analysis

A multivariate ANOVA statistical analysis was performed on the results obtained, together with a WILKS test and the F test. A homogeneous group was assumed with alpha 0.005 and df = 10,000.

## 3. Results

### 3.1. Weight Loss

The results of biomass weight loss measurements following steam explosion pretreatment are presented in Table 1.

The smallest weight loss was obtained at a temperature of 160 °C and amounted to 5.3%. The largest weight loss was obtained at a process temperature of 205 °C. It was slightly lower at a temperature of 190 °C.

### 3.2. Metal Content After Steam Explosion

Table 1 and Table 2 show the results of the content of metals considered to be inhibitors of biological processes. Table 2 shows the content of metals determined in wood incinerated before pre-treatment and after pretreatment by steam explosion. The results for Cr, Fe, Ni, Cu, Mn, and Zn metal content are presented in g/g. The standard deviation for the results is also presented.

The analysis of metal content in wood biomass after SE pretreatment in incinerated material, we can observe that the element present in the largest amount is zinc for the experiment conducted at a temperature of 175 °C, amounting to almost 6. 5 × 10^3^ g/g. The lowest content of this element was obtained for the native material and amounts to almost 1 × 10^3^ g/g. Another element with a significant increase is iron, the highest concentration of which was obtained for pre-treatment at a temperature of 175 °C and amounted to almost 6 × 10^3^ g/g. The content of all metals except manganese is highest for biomass after pre-treatment at a temperature of 175 °C. However, the highest manganese content determined in ash was obtained for the native material and amounted to almost 2 × 10^3^ g/g.

The analysis of metal content in dissolved ash is presented in Table 3. The highest content was determined for zinc in biomass after treatment at 175 °C, amounting to almost 0.9 × 10^3^ g/g. The highest concentrations of iron, nickel and copper were also determined at this temperature, amounting to almost 0.2 × 10^3^ g/g, 0.04 × 10^3^ g/g and 0.03 × 10^3^ g/g, respectively. For chromium and manganese, the highest content was observed for the native material and amounted to 0.01 × 10^3^ g/g and 0.03 × 10^3^ g/g, respectively.

The metal content determined after dissolving the ash in nitric acid showed different values than those found in the ash itself. The trend in the content of individual metals in the material is the same for both assay variants. A detailed graphical representation of the results is presented in the Discussion section, where the obtained results are compared with the findings of other researchers.

The results of the metal content in the material after steam explosion treatment indicate ambiguous behavior of the tested elements. The highest content of metal inhibitors is observed at 175 °C. At this temperature, iron, nickel, zinc, and copper exhibit the highest content. The content of potential metallic inhibitors in the material after the steam explosion at 205 °C is the lowest except for zinc where the value is higher than in the native material and after the process at 160 °C. In the case of chromium and manganese, native wood shows the highest levels of these elements. Chromium is most abundant outside the native material in the wood after the SE process at 190 °C.

### 3.3. Statistical Analysis

A basic multivariate ANOVA statistical analysis was performed on the obtained data, together with Wilks’ test. The analysis allowed four groups to be identified, to which the data from Table 2 and Table 3 were assigned.

The results obtained for charred wood were as follows: Wilks’ lambda obtained was 0.00032, the F test result was 7.0531, and the *p*-value was 0.00003. The results obtained for wood that was incinerated and then dissolved in nitric acid were as follows: Wilks’ lambda obtained for chromium Cr was 0.0000, the F test result was 15.0522, and the *p* value was 0.00000.

## 4. Discussion

### 4.1. Weight Loss

Analysing the data obtained and presented in Table 1 during pretreatment with a steam explosion, the mass of the material on which the process takes place is reduced. In the study presented here, a weight loss of up to 11.5% was achieved. This value depends on the process temperature and the biomass being treated [31,32]. The value of this process is most effective at temperatures above 240 °C [31,33].

This is a result of the thermal strength of the biomass components. The least resistant structural components are hemicelluloses, with amorphous lignin decomposing at higher temperatures, followed by cellulose, which can withstand 400 °C [34].

The results obtained for weight loss due to pretreatment are consistent with the data in the literature.

### 4.2. Metal Content After Steam Explosion

**Figure 1:**
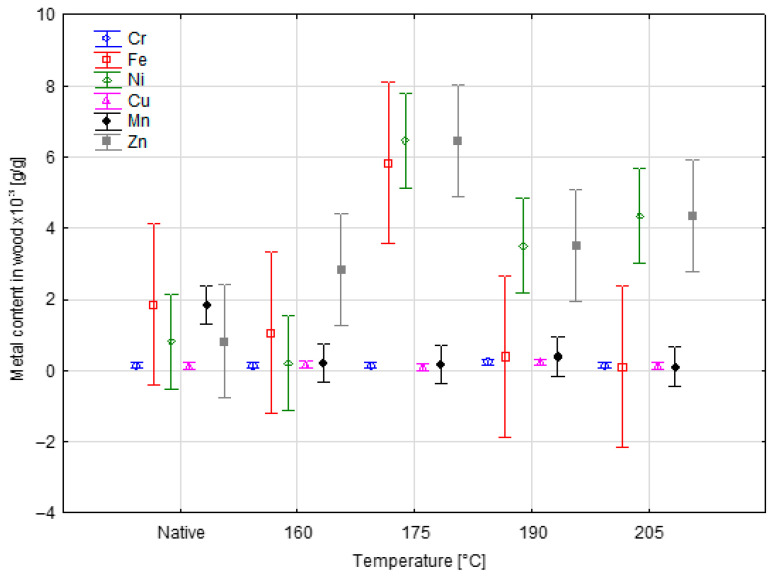
Metal content in wood measured in ash after steam explosion.

**Figure 2:**
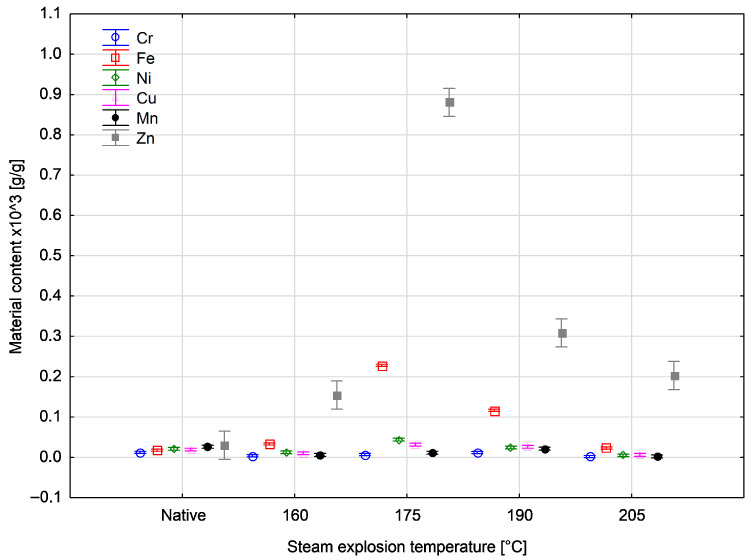
Changes in the content of metallic elements measured after dissolving ash in acid as a function of temperature.

The results of Table 2 and Table 3 are presented in Figure 1 and Figure 2, respectively, in order to better illustrate the changes in metal content resulting from pretreatment with steam explosion.

The measurement of elemental content depends on the method and the material being analyzed. Wood is one of the most difficult materials to analyze due to its non-uniform structure and differences in density [35].

Zielenkiewicz et al. [30] pointed out in their work that the key to determining accurate metal content in woody biomass using XRF is to calibrate the method to the specific wood species being analysed. The method of analysing samples after ashing is more accurate than analysing unashed wood but is not free of drawbacks. The most accurate results are obtained for ash dissolved in nitric acid [29]. The results obtained during the realisation of our work show a high variability in ashed versus ashed and dissolved material in nitric acid. The problem of accurately determining the metal content of non-metallic samples can arise from the heterogeneous structure of the material being analysed [36,37,38,39].

**Figure 3:**
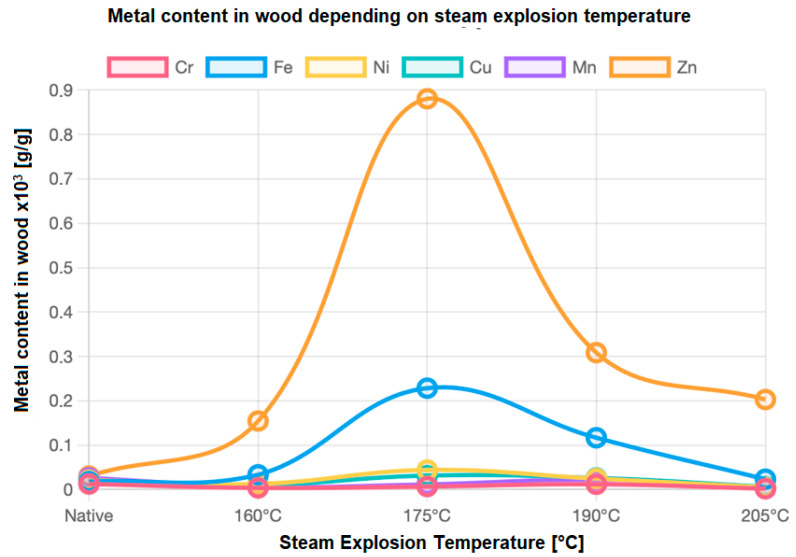
Distribution of metal content in wood depending on the temperature of steam explosion.

Our own research has shown that the distribution of metal content in wood during variable steam explosion temperatures is similar to normal distribution (Figure 3). The highest peak was recorded for zinc at a temperature of 175 °C.

Krutul et al. [40] indicate that the heavy metal content of poplar biomass due to pretreatment processes such as steam explosion (SE) and liquid hot water (LHW) increases with the temperature of the treatment processes as well as with the duration of the holding time of the annealing, the summed results of the metal content for liquid and solid biomass show the highest metal content for processes at 175 °C and holding time of 60 min. The determined metal contents of the analysed material should not affect the biological processes of the industry. However, the problem of metal ion contamination due to industrial impacts is a significant and increasingly important issue [16,41].

Unlike organic substances, metal contaminants are not subject to natural decomposition. Heavy metals in the form of ions, on the other hand, are accumulated in the environment, leading to severe environmental contamination [42,43,44,45]. In addition, the metal content in wood is influenced by the area of the trunk from which the analysed material originates, the place of growth (metallic contaminants that the wood may have absorbed during growth), and any contaminants resulting from processing (contaminants from cutting tools) [46,47,48].

The permissible iron content in drinking water according to the Regulation of the Minister of Health of the Republic of Poland of 7 December 2017 is up to 200 µg/L, for manganese it is up to 50 µg/L, cyanide up to 50 µg/L, the content is not specified but is usually below 0.002 to 0.006 mg/L in drinking water, copper up to 2.0 mg/L, nickel up to 20 µg/L [49]. The above limits are in accordance with Directive (EU) 2020/2184 of the European Parliament and of the Council of 16 December 2020 on the quality of water intended for human consumption [50].

### 4.3. Statistical Analysis

The results of statistical analysis of the obtained results indicate significant variability for the obtained values depending on the temperature of the pre-treatment process using steam explosion. Performing measurements on incinerated material and then dissolving it in nitric acid leads to a decrease in the values of *p* and Wilks’ lambda, as well as an increase in the value of F. This indicates a standardization of materials within groups, leading to a sharpening of the relationship between individual pretreatment temperatures [2,6,8,29].

## 5. Conclusions

Investigations of the heavy metal content of steam-explosion-treated GM poplar ash recorded the following observations and conclusions:

(1) The highest metallic inhibitor content was usually observed at 175 °C. This contamination, although minor, may be of importance when using a closed water circuit due to the accumulation of metallic inhibitors in the effluent. Future studies should investigate whether enzymatic hydrolysis carried out on material contaminated with metallic inhibitors to this extent will have lower efficiency.

(2) XRF testing is a simple and rapid method for investigating the content of metallic elements and the change in their occurrence in the materials under study, which gives the best results for comparative measurements.

(3) Dissolution in acid gives a more accurate comparison of values and the truest measurement results due to the homogenisation of the sample and a reduction in measurement uncertainty. The disadvantage of this method is the use of concentrated nitric acid and its neutralisation with ammonia water, accompanied by the release of energy in the form of heat, so it is unsafe. In addition, dissolving the ash leads to a ‘dilution’ of the metals under test.

(4) The process of steam explosion in most of the cases studied results in “washing” of the metals under investigation out of the apparatus. However, it depends on the temperature used, to what extent these metals penetrate into the solid phase that would be subjected to enzymatic hydrolysis after pretreatment. From the point of view of the inhibitory effect of metals in the process, the lowest temperature (160 °C) or the highest temperature (205 °C) of those proposed should be considered.

(5) The liquid phase should also be taken into account during future studies of the heavy metal content of the material after the steam explosion. Although it is the solid phase that is the element of further processing and the metal content in this phase seems to be the most important, determining their content also in the liquid phase can provide a lot of valuable information about the phenomena occurring in the reactor.

## Figures and Tables

**Table 1:** Loss of wood biomass due to a steam explosion.

Steam Explosion Temperature [°C]	Average Weight of Dry Samples Before SE [g]	Mass of Wet Chips After SE [g]	Dry Shavings Weight After Draining [g]	Material Weight Loss [g]	Material Weight Loss [%]
160	18.988	42.82	17.984	1.003	5.3
175	18.892	27.83	16.976	1.915	10.1
190	18.926	32.23	16.760	2.166	11.4
205	18.894	22.3	16.725	2.169	11.5

**Table 2:** Metal content in wood measured in ash.

Temperature [°C]	Sample	Metal Content in Wood × 10^3^ [g/g]					
		Cr	Fe	Ni	Cu	Mn	Zn
160	Average	0.14392	1.07192	0.20548	0.16069	0.19723	2.84 197
	Standard deviation (SD)	0.06235	0.40234	0.07452	0.05627	0.03717	1.47 619
175	Average	0.14569	5.83317	6.46632	0.09413	0.15745	6.46632
	SD	0.05953	3.79896	1.03646	0.10620	0.01812	1.03646
190	Average	0.22880	0.39063	3.50958	0.22880	0.39063	3.50958
	SD	0.06827	0.06474	1.05307	0.06827	0.06474	1.05307
205	Average	0.14129	0.11287	4.36040	0.14129	0.11287	4.36040
	SD	0.05123	0.02576	1.68736	0.05123	0.02576	1.68736
Nativ	Average	0.14271	1.84917	0.82471	0.14271	1.84917	0.82471
	SD	0.06428	0.93937	0.55677	0.06428	0.93937	0.55677
	Group 1—correlation occurs to a large extent—close correlation						
	Group 2—moderate correlation						
	Group 3—correlation occurs to a small extent						

**Table 3:** Metal content in wood measured after dissolving the ash in acid.

Temperature [°C]	Sample	Metal Content in Wood × 10^3^ [g/g]					
		Cr	Fe	Ni	Cu	Mn	Zn
160	Average	0.00369	0.03320	0.01274	0.01017	0.00634	0.15440
	SD	0.00026	0.00039	0.00034	0.00021	0.00014	0.00156
175	Average	0.00675	0.22837	0.04405	0.03150	0.01153	0.88052
	SD	0.00135	0.00282	0.00240	0.00242	0.00196	0.00519
190	Average	0.01181	0.11675	0.02461	0.02615	0.02179	0.30847
	SD	0.00366	0.00249	0.00358	0.00246	0.00488	0.06003
205	Average	0.00189	0.02330	0.00483	0.00635	0.00254	0.20284
	SD	0.00039	0.00129	0.00089	0.00085	0.00069	0.00446
Nativ	Average	0.01246	0.01803	0.02128	0.01939	0.02629	0.03029
	SD	0.00277	0.00254	0.00415	0.00482	0.00377	0.00536
	correlation occurs to a large extent—close correlation						
	no labelling, no correlation observed						

## Data Availability

The original contributions presented in this study are included in the article. Further inquiries can be directed to the corresponding authors.

## References

[1] Szadkowski J., Radomski A., Antczak A., Szadkowska D., Lewandowska A., Marchwicka M., Kupczyk A. (2017). The yield of model hydrolysis and fermentation in the technology of bioethanol production from poplar wood (*Populus* sp.). Przemysł Chem..

[2] Roman K., Barwicki J., Hryniewicz M., Szadkowska D., Szadkowski J. (2021). Production of Electricity and Heat from Biomass Wastes Using a Converted Aircraft Turbine AI-20. Processes.

[3] Segurado R., Pereira S., Correia D., Costa M. (2019). Techno-economic analysis of a trigeneration system based on biomass gasification. Renewable and Sustainable Energy Reviews. April.

[4] Prosiński S. (1984). Chemia Drewna.

[5] Szadkowska D., Auriga R., Lesiak A., Szadkowski J., Marchwicka M. (2022). Influence of Pine and Alder Woodchips Storage Method on the Chemical Composition and Sugar Yield in Liquid Biofuel Production. Polymers.

[6] Roman K. (2025). The Estimation of the Possibility of Bioethanol Production from Hemp Cellulose Using the HWE Method. Energies.

[7] Marchwicka M., Antczak A., Drożdżek M., Akus-Szylberg F., Szadkowska D., Szadkowski J., Żmuda E., Radomski A., Zawadzki J. (2023). Influence of selected treatment methods on the dry residue and sugars content extracted from wheat and rye bran. Ann. Wars. Univ. Life Sci. SGGW. For. Wood Technol..

[8] Roman K., Dasiewicz J., Marchwicka M. (2024). Impact of Hot Water Extraction on the Compaction Efficiency and Material Properties of *Miscanthus giganteus* in Pellet Production. Materials.

[9] Krutul D., Szadkowski J., Výbohová E., Kučerová V., Čabalová I., Antczak A., Szadkowska D., Drożdżek M., Zawadzki J. (2024). Effect of steam explosion pretreatment on chosen saccharides yield and cellulose structure from fast-growing poplar (*Populus deltoides* × *maximowiczii*) wood. Wood Sci. Technol..

[10] Yildiz S., Gümüşkaya E. (2007). The effects of thermal modification on crystalline structure of cellulose in soft and hardwood. Build. Environ..

[11] Yang B., Dai Z., Ding S.Y., Wyman C.E. (2011). Enzymatic hydrolysis of cellulosic biomass. Biofuels.

[12] Tomás-Pejó E., Alvira P., Ballesteros M., Negro M.J., Pandey A., Larroche C., Ricke S.C., Dussap C.-G., Gnansounou E., Biofuels (2011). Chapter 7—Pretreatment Technologies for Lignocellulose-to-Bioethanol Conversion.

[13] Karimi K., Taherzadeh M.J. (2016). A critical review of analytical methods in pretreatment of lignocelluloses: Composition, imaging, and crystallinity. Bioresour. Technol..

[14] Kačík F., Kačíková D., Jablonský M., Katuščák S. (2009). Cellulose degradation in newsprint paper ageing. Polym. Degrad. Stab..

[15] Gałązka A., Szadkowski J. (2021). Enzymatic Hydrolysis of Fast-Growing Poplar Wood After Pretreatment by Steam Explosion. Cellul. Chem. Technol..

[16] Czatzkowska M., Harnisz M., Korzeniewska E., Koniuszewska I. (2020). Inhibitors of the methane fermentation process with particular emphasis on the microbiological aspect: A review. Energy Sci. Eng..

[17] Sun X.-Y., Zhao Y., Liu L.-L., Jia B., Zhao F., Huang W.-D., Zhan J.-C. (2015). Copper Tolerance and Biosorption of *Saccharomyces cerevisiae* during Alcoholic Fermentation. PLoS ONE.

[18] Lindholm-Lehto P.C. (2019). Biosorption of Heavy Metals by Lignocellulosic Biomass and Chemical Analysis. Biomass metal adsorption. BioResources.

[19] Milne T., Brennan A.H., Glenn B.H. (1990). Sourcebook of Methods of Analysis for Biomass and Biomass Conversion Processes. Elsevier Applied Sciences.

[20] Li Y., Zhou L.W., Wang R.Z. (2017). Urban biomass and methods of estimating municipal biomass resources. Renew. Sustain. Energy Rev..

[21] Pastircakova K. (2004). Determination of trace metal concentrations in ashes from various biomass materials. Energy Educ. Sci. Technol..

[22] Debela F., Thring R.W., Arocena J.M. (2012). Immobilization of Heavy Metals by Co-pyrolysis of Contaminated Soil with Woody Biomass. Water Air Soil Pollut..

[23] Giudicianni P., Gargiulo V., Grottola C.M., Alfè M., Ferreiro A.I., Almeida Mendes M.A., Fagnano M., Ragucci R. (2021). Inherent Metal Elements in Biomass Pyrolysis: A Review. Energy Fuels.

[24] Sun Y., Cheng J.J. (2002). Hydrolysis of lignocellulosic materials for ethanol production: A review. Bioresour. Technol..

[25] Bauer A., Bosch P., Friedl A., Amon T. (2009). Analysis of methane potentials of steam-exploded wheat straw and estimation of energy yields of combined ethanol and methane production. J. Biotechnol..

[26] Holtzapple M.T., Humphrey A.E., Taylor J.D. (1989). Energy requirements for the size reduction of poplar and aspen wood. Biotechnol. Bioenergy.

[27] Li X., Zhang N.N., Quyang J., Xu Y., Yong Q.A., Yu S.Y., Sun R.C., Fu S.Y. (2010). Optimization of steam-pretreatment conditions for corn stover using response surface methodology, Conference Paper. Research Progress in Paper Industry and Biorefinery (4TH ISETPP).

[28] Kubicek C.K. (2013). Fungi and Lignocellulosic Biomass.

[29] Zielenkiewicz T. (2015). Nowe metody analizy instrumentalnej wybranych pierwiastków i związków chemicznych w drewnie i kompozytach drzewnych [EN: New methods of instrumental analysis of chosen elements and chemical compounds in wood and wood composites]. Rozprawy Naukowe.

[30] Zielenkiewicz T., Zawadzki J., Radomski A. (2012). XRF spectrometer calibration for copper determination in wood. X-Ray Spectrom..

[31] Tanase-Opedal M., Ghoreishi S., Hermundsgård D.H., Barth T., Moe S.T., Brusletto R. (2024). Steam explosion of lignocellulosic residues for co-production of value-added chemicals and high-quality pellets. Biomass Bioenergy.

[32] Akizuki S., Suzuki H., Fujiwara M., Toda T. (2023). Impacts of steam explosion pretreatment on semi-continuous anaerobic digestion of lignin-rich submerged macrophyte. J. Clean. Prod..

[33] Ziegler-Devin I., Chrusciel L., Brosse N. (2021). Steam Explosion Pretreatment of Lignocellulosic Biomass: A Mini-Review of Theorical and Experimental Approaches. Front. Chem..

[34] Zhu J., Guo Y., Chen N., Chen B. (2024). A Review of the Efficient and Thermal Utilization of Biomass Waste. Sustainability.

[35] Akinyele O.S., Shokunbi I.O. (2015). Comparative analysis of dry ashing and wet digestion methods for the determination of trace and heavy metals in food samples. Food Chem..

[36] Kalnicky D.J., Singhvi R. (2001). Field portable XRF analysis of environmental samples. J. Hazard. Mater..

[37] Trojek T., Dušková A. (2024). Quantitative X-ray fluorescence micro-analysis of wood samples and visualization of tree rings. Radiat. Phys. Chem..

[38] Scharnweber T., Rocha E., González Arrojo A., Ahlgrimm S., Gunnarson B.E., Holzkämper S., Wilmking M. (2023). To extract or not to extract? Influence of chemical extraction treatment of wood samples on element concentrations in tree-rings measured by X-ray fluorescence. Front. Environ. Sci..

[39] Block C.N., Shibata T., Solo-Gabriele H.M., Townsend T.G. (2007). Use of handheld X-ray fluorescence spectrometry units for identification of arsenic in treated wood. Environ. Pollut..

[40] Krutul D., Szadkowski J., Antczak A., Drożdżek M., Radomski A., Karpiński S., Zawadzki J. (2021). The Concentration of Selected Heavy Metals in Poplar Wood Biomass and Liquid Fraction Obtained after High Temperature Pretreatment. Wood Res..

[41] Mueller R.F., Steiner A. (1992). Inhibition of Anaerobic Digestion Caused by Heavy Metals. Water Sci. Technol..

[42] Chandel A.K., da Silva S.S., Singh O.V. (2013). Detoxification of Lignocellulose Hydrolysates: Biochemical and Metabolic Engineering Toward White Biotechnology. Bioenergy Res..

[43] Galvagno S., Gasciaro G., Casu S., Martino M., Mingazzini C., Russo A., Portofino S. (2009). Steam gasification of tyre waste, poplar, and refuse-derived fuel: A comparative analysis. Waste Manag..

[44] Xu Q., Li X., Ding R., Wang D., Liu Y., Wang Q., Zhao J., Chen F., Zeng G., Yang Q. (2017). Understanding and mitigating the toxicity of cadmium to the anaerobic fermentation of waste activated sludge. Water Res..

[45] Li C., Fang H.P. (2007). Inhibition of heavy metals on fermentative hydrogen production by granular sludge. Chemosphere.

[46] Zielenkiewicz T., Szadkowski J., Drożdżek M., Zielenkiewicz A., Kłosińska T., Antczak A., Zawadzki J., Gawron J. (2016). Application of X-Ray Fluorescence Technique for Determination of Heavy Metals Uptake by Different Species of Poplar. Drewno.

[47] Krutul D., Zielenkiewicz T., Zawadzki J., Radomski A., Antczak A., Drożdżek M. (2018). Influence of Urban Agglomeration Environmental Pollution on Content of Chosen Metals in Bark, Roots and Wood of Norway Maple (*Acer Platanoides* L.). Wood Res..

[48] Zawadzki J., Zielenkiewicz T., Radomski A., Witomski P., Drożdżek M. (2010). Testing Content of Copper in Scots Pine Wood (*Pinus Sylvestris* L.) after Preservative Treatment. Wood Res..

[49] Rozporządzenie Ministra Zdrowia z Dnia 7 Grudnia 2017 r. w Sprawie Jakości Wody Przeznaczonej do Spożycia Przez Ludzi (Dz.U. 2017, poz. 2294). [Regulation of the Minister of Health of 7 December 2017 on the Quality of Water Intended for Human Consumption (Journal of Laws 2017, Item 2294)]. https://isap.sejm.gov.pl/isap.nsf/download.xsp/WDU20170002294/O/D20172294.pdf.

[50] Directive (EU) 2020/2184 of the European Parliament and of the Council of 16 December 2020 on the Quality of Water Intended for Human Consumption (Recast) (Text with EEA Relevance). https://eur-lex.europa.eu/legal-content/EN/TXT/PDF/?uri=CELEX:32020L2184.

